# Bayesian Update with Information Quality under the Framework of Evidence Theory

**DOI:** 10.3390/e21010005

**Published:** 2018-12-21

**Authors:** Yuting Li, Fuyuan Xiao

**Affiliations:** School of Computer and Information Science, Southwest University, Chongqing 400715, China

**Keywords:** Bayesian update, information quality, Dempster-Shafer evidence theory, basic probability assignment, target recognition, prior probability distribution, posterior probability distribution

## Abstract

Bayesian update is widely used in data fusion. However, the information quality is not taken into consideration in classical Bayesian update method. In this paper, a new Bayesian update with information quality under the framework of evidence theory is proposed. First, the discounting coefficient is determined by information quality. Second, the prior probability distribution is discounted as basic probability assignment. Third, the basic probability assignments from different sources can be combined with Dempster’s combination rule to obtain the fusion result. Finally, with the aid of pignistic probability transformation, the combination result is converted to posterior probability distribution. A numerical example and a real application in target recognition show the efficiency of the proposed method. The proposed method can be seen as the generalized Bayesian update. If the information quality is not considered, the proposed method degenerates to the classical Bayesian update.

## 1. Introduction

Probability is one of the often used tools to deal with uncertainty [1]. In probability theory, Bayesian method occupies an important position. Bayesian method has been widely used in various aspects, such as artificial intelligence [2,3], pattern recognition [4], spam filtering [5], construct and estimate biology models [6], Chinese word segmentation and semantics [7,8], exoplanetary explore [9,10,11], Multi-criteria decision making [12,13] and others [14,15,16,17].

Bayesian update is a popular topic [18,19], Vinogradova proposed using Bayes methods to recalculate the weight [20]. However, there are some situations in which the classical Bayesian method cannot cope with information fusion intuitively. If the probability distributions exist with high degree conflict, classical Bayes methods are no longer applicable. Recently, Yager proposed a new information quality (IQ) [21], quality is related to the lack of uncertainty in the fused value and the use of credible sources, information quality is a measure of information or certainty. The value provides us a method to evaluate the probability distribution. Inspired by Yager and Vinogradova, we decided one possible way to address this issue is to take the information quality into consideration. The main contribution of this paper is proposed a new Bayesian update method with the information quality based on the framework of evidence theory.

Dempster-Shafer evidence theory plays an important role in intelligent system [22,23]. There are two main advantages. One is the better ability to model uncertain information [24,25,26,27,28]. Information fusion enables us to get more useful information from a large amount of data, which is another advantage of evidence theory [29,30]. The Dempster’s combination rule has the ability to combine a variety of source information in a straight way [31,32,33]. Some research argues that Bayesian method is a special case of Dempster-Shafer evidence theory [34,35]. When the basic probability assignment (BPA) is only assigned to a single subset, Dempster’s combination rule degenerates to Bayesian law.

The motivation of this paper is to improve the ability of the classical Bayesian rule to deal with highly conflicting information. As illustrated in some examples in the following sections, the counterintuitive result is obtained with classical Bayesian method. To address this issue, a new Bayesian update with information quality under the framework of evidence theory is presented. Based on the framework of Dempster-Shafer evidence theory, the novel Bayesian update method is proposed. The information quality of the prior probability distribution is taken as weight and the discounting coefficient is determined by the weight. Then, the basic probability assignment can be obtained from prior probability distribution with the discounting coefficient. Next, the combination result of basic probability assignment from different sensor reports is combined by using Dempster’s combination rule. Finally, the pignistic probability distribution transformation is used to obtain the posterior probability.

The rest of this paper is organized as follows. Section 2 introduces the preliminary knowledge. Section 3 presents the method to deal with Bayesian update based on information quality. Section 4 illustrates the use of the proposed method in target recognition. Section 5 is a brief summary of this article.

## 2. Preliminaries

This section will introduce some preliminary knowledge regarding evidence theory [36,37], the pignistic probability transformation [38] and information quality [21].

### 2.1. Evidence Theory

There are many methods to cope with uncertainty information, such as Z-numbers [39,40], fuzzy set [41,42,43,44,45], grey theory [46,47,48], D-numbers [49,50,51,52,53]. Dempster-Shafer evidence theory has been widely used, such as in risk assessment [54], environment management [55], fault diagnosis [56,57], and decision making [58,59]. Assume Ω is the frame of discernment. The components of Ω are infinite nonempty sets with the attributes of mutually exclusive and exhaustive. Let the components of $\Omega =\{\emptyset ,A_{1},A_{2},\cdots ,A_{n}\}$. The power set of Ω is $2^{\Omega }$ equal to $2^{n}$. Each component of the power set is a subset of Ω.

**Definition** **1.** *The basic probability assignment (BPA) is a mapping $m:2^{\Omega }\to \left[0,1\right]$ that satisfies [36,37]:*

(1) $$m\left(\emptyset \right)=0\;and\;\sum_{A\subseteq \Omega }m\left(A\right)=1,$$

*where A is a subset of* Ω.

**Definition** **2.** *Given two basic probability assignments, Dempster’s combination rule is defined as follows [36,37],*

(2) $$m\left(C\right)=m_{i}\left(X\right)⊕m_{i^{\prime }}\left(Y\right)=\left\{\begin{array}{cc} 0 & ifX\cap Y=\emptyset \\ \frac{\sum_{X\cap Y=C,\forall X,Y\subseteq \Omega }m_{i}\left(X\right)*m_{i^{\prime }}\left(Y\right)}{1-k} & ifX\cap Y\neq \emptyset \end{array}\right.$$

*with k shows the conflict among the collected evidence, defined as follows,*

(3) $$k=\sum_{X\cap Y=\emptyset ,\forall X,Y\subseteq \Omega }m_{i}\left(X\right)*m_{i^{\prime }}\left(Y\right)$$

However, when the value of *k* is big, use of Dempster’s combination rule will produce counter intuitive results [60,61]. Yager [62], Dubois [63] and Smets [64] and more recently Murphy [65], Deng et al. [66] and other researchers [67] have proposed alternative combination rules. Recently, with the belief entropy [68,69], some new methods are presented to address the conflict [70,71,72].

The next example illustrates how the Bayesian update cannot cope with high degree conflict probability distributions.

**Example** **1.** *Using the classical Bayesian method to deal with the high degree conflict example, the probability distribution is $p_{1}$: (0.9900, 0.0100, 0), $p_{2}$: (0, 0.0100, 0.9900).*

k=0.9900∗0.0100+0.9900∗0.9900+0.9900∗0.0100=0.9999

$$m\left(A\right)=m\left(C\right)=\frac{0}{1-0.9999}=0$$

$$m\left(B\right)=\frac{0.0001}{1-0.9999}=1$$

*So the final combination result is P: (0, 1, 0).*

It is obvious that the combination result is counterintuitive. The example shows the classical Bayesian method cannot update the probability distribution when conflict exists, but the proposed method can update the conflicting probability distributions. Details for the combination steps are given in Example 6.

### 2.2. Pignistic Probability Transformation

Pignistic probability transformation (PPT) is used to transfer basic probability assignment into probability distribution, defined as follows [38],

**Definition** **3.** *Let m be a basic probability assignment on* Ω *and its associated pignistic probability transformation $BetP_{m}$: Ω→[0,1] is defined as [38]*

(4) $$BetP_{m}\left(\omega \right)=\sum_{A\subseteq \Omega ,\omega \epsilon A}\frac{1}{|A|}\frac{m(A)}{1-m(\emptyset )}\;m\left(\emptyset \right)\neq 1$$

*with ∣A∣ being the cardinality of subset A.*

**Example** **2.** *Let one BPA from distinct sources on frame $\Omega =\{\omega_{1},\omega_{2},\omega_{3},\omega_{4}\}$ be*

$m_{1}\left(\left\{\omega_{1},\omega_{2}\right\}\right)=0.8000$   $m_{1}\left(\left\{\omega_{3}\right\}\right)=0.1000$   $m_{1}\left(\left\{\omega_{4}\right\}\right)=0.1000$
*Let B = $\left\{\omega_{1}\right\}$ then $BetP_{1}\left(B\right)=0.4000$*
*Let A = $\left\{\omega_{4}\right\}$ then $BetP_{1}\left(A\right)=0.1000$*

### 2.3. Information Quality

Entropy is a measure of uncertainty associated with the information [73]. More uncertainty means more entropy; the smaller the entropy, the more information is contained in this probability distribution. There are several methods to calculate entropy [74,75], some famous entropies are as follows, Shannon entropy [76], Gini entropy [77], Deng entropy [78,79] and others [80].

**Definition** **4.** *Gini entropy is defined as follows [77],*

(5) $$G\left(p_{i}\right)=1-\sum_{i=1}^{n}{∥p_{i}∥}^{2}$$

*where $p_{i}$ is the vector form of probability distribution.*

From the definition of Gini entropy, it is obvious that in order to magnify the value of $G(p_{i})$ the value of $\sum_{i=1}^{n}{∥p_{i}∥}^{2}$ the bigger the better. For this reason, Yager proposed use of ${∥p_{i}∥}^{2}$, named NegEnt, as a measure of information or certainty [21]. Information quality has been applied in decision making [81,82], evaluating information [26], in maximal fusion [83,84], modeling [85] and elsewhere [24,86].

The bigger the NegEnt (${∥p_{i}∥}^{2}$), the smaller the entropy, the more certainty provided by the probability distribution; the information increases by increasing NegEnt (${∥p_{i}∥}^{2}$).

**Definition** **5.** *Given a probability distribution $p_{i}$, the information quality is defined as follows [21],*

(6) $${IQ}_{p_{i}}={∥p_{i}∥}^{2}=\sum_{j=1}^{n}{\left(p_{ij}\right)}^{2}$$

*while $∥p_{i}∥$ is defined as follows [21],*

(7) $$∥p_{i}∥=\sqrt{\left(p_{i}\right)*\left(p_{i}\right)}={\left(\sum_{j=1}^{n}{\left(p_{ij}\right)}^{2}\right)}^{\frac{1}{2}}$$

**Example** **3.** *Given a probability distribution p: (0.3000,0.6000,0.1000), the corresponding information quality can be calculated as*

$${IQ}_{p_{i}}=0.3000*0.3000+0.6000*0.6000+0.1000*0.1000=0.4600$$

## 3. Proposed Method

This section first describes the method to determine weight, based on the information quality of the probability distribution. Then a new method is presented to generate basic probability assignment based on the weight. Next, Dempster’s combination rule is used to fuse basic probability assignments. Finally, with the aid of PPT, the fusion transformation to probability distribution is detailed.

### 3.1. Determine Weight

The information quality is an important index to measure the quality of the information. The weights of the probability distributions quantitatively express their significance and influence the evaluation result [87], so it is reasonable to take information quality as the weight of the probability distribution. The information quality needs to be normalized since the sum of the weights must meet the attribute, the summation of the weight must equal to one. The weight can be seen as a discounting coefficient and we can use it to generate basic probability distribution.

**Definition** **6.** *Given the probability distribution $p_{i}$, the corresponding weight is defined as follows,*

(8) $$\omega_{i}=\frac{{IQ}_{p_{i}}}{{\sum_{i}}^{n}{IQ}_{p_{i}}}$$

*where ${IQ}_{p_{i}}$ is the information quality of the probability distribution.*

**Example** **4.** *If the information quality is given as follows, ${IQ}_{p_{1}}=0.5400$, ${IQ}_{p_{2}}=0.8200$, ${IQ}_{p_{3}}=0.6600$.*

*Then the corresponding weighting can be calculated as follows,*

$$\omega_{p_{1}}=\frac{0.5400}{0.5400+0.8200+0.6600}=0.2700$$

$$\omega_{p_{2}}=\frac{0.8200}{0.5400+0.8200+0.6600}=0.4000$$

$$\omega_{p_{3}}=\frac{0.6600}{0.5400+0.8200+0.6600}=0.3300$$

### 3.2. Generate Basic Probability Assignent

This section proposed the method to convert a probability distribution to a basic probability assignment, based on the weight of the probability distribution. Note, there is a one-to-one correspondence between probability distribution and basic probability assignment. Algorithm 1 illustrates the method to get basic probability assignment.
**Algorithm 1:** The algorithm to generate a basic probability assignment// To get all BPA, execute this algorithm n (total number of probability distributions) times as the algorithm is used to convert a probability distribution to a BPA. **Input**: The weight of the probability distribution, $\omega_{1}$ $m\left(A\right)=\omega_{1}*p\left(A\right)$ $m\left(B\right)=\omega_{1}*p\left(B\right)$ ⋯ $m\left(N\right)=\omega_{1}*p\left(N\right)$ $m\left(AB\cdots N\right)=1-\sum_{i=1}^{n}\omega_{1}*p\left(I\right)\;\;I=A,B,C,\cdots ,N$ **Output**: $m_{1}=\left(\left\{m\left(A\right)\right\},\left\{m\left(B\right)\right\},\left\{\cdots \right\},\left\{m\left(AB\cdots N\right)\right\}\right)$

### 3.3. Fusion Method

This section shows how to combine basic probability assignment multiple times and how to transform the fusion result into a probability distribution.

Only two basic probability assignments are involved in fusion at any time. The first and the second basic probability assignment participate the fusion first. Then the fusion result and the next basic probability assignment are involved in fusion, and the rest are fused in turn until all the BPA are involved in the fusion. The pseudo-code in Algorithm 2 illustrates the fusion process intuitively.
**Algorithm 2:** The algorithm of fusion process
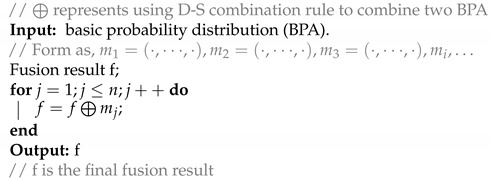


**Figure 1:**
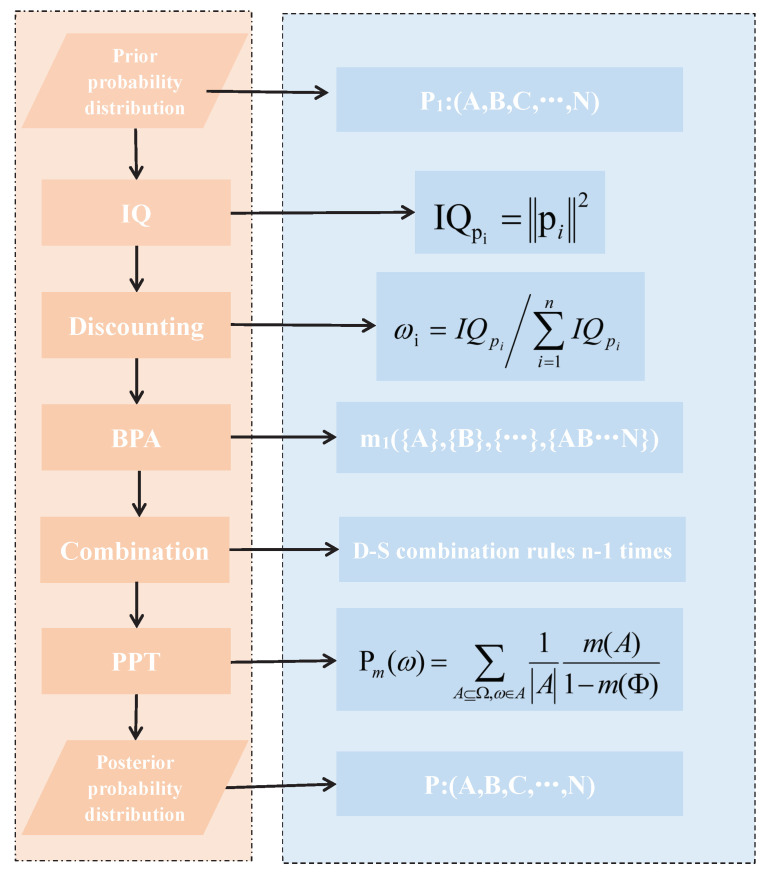
Flow chart of the presented method.

Next, a flow chart (Figure 1) illustrates the whole process of the proposed method.

As can be seen in Figure 1, the additions of the previous works are mainly in two aspects: first, the information quality is taken into account in the process of the Bayesian update. Second, the Bayesian update proposed in this paper is based on the framework of the evidence theory.

## 4. Application

In this section, a numerical example will first illustrate the use of the proposed method. Example 1 is then revisited with the use of the new approach. Finally, two real applications in target recognition demonstrates how the proposed method can be applied.

### 4.1. Numerical Example

This numerical example shows the process of the proposed method.

**Example** **5.** *The probability distributions are $p_{1}$: ($\frac{1}{3}$, $\frac{1}{3}$, $\frac{1}{3}$), $p_{2}$: (0.7000, 0.2000, 0.1000), $p_{3}$: (0.6000, 0.3000, 0.1000).*

*The corresponding information qualities are:*

$${IQ}_{p_{1}}=0.3300$$

$${IQ}_{p_{2}}=0.5400$$

$${IQ}_{p_{3}}=0.4600$$

*The corresponding weightings are:*

$$\omega_{p_{1}}=\frac{0.3300}{0.3300+0.5400+0.4600}=0.2500$$

$$\omega_{p_{2}}=\frac{0.5400}{0.3300+0.5400+0.4600}=0.4000$$

$$\omega_{p_{3}}=\frac{0.4600}{0.3300+0.5400+0.4600}=0.3500$$

*The generated basic probability assignments are:*

$$m_{1}=\left(\left\{\frac{1}{12}\right\},\left\{\frac{1}{12}\right\},\left\{\frac{1}{12}\right\},\left\{\frac{3}{4}\right\}\right)$$

$$m_{2}=\left(\left\{0.2800\right\},\left\{0.0800\right\},\left\{0.0400\right\},\left\{0.6000\right\}\right)$$

$$m_{3}=\left(\left\{0.2000\right\},\left\{0.1100\right\},\left\{0.0400\right\},\left\{0.6500\right\}\right)$$

*Next, use of Dempster’s combination rule 2 times gives the following.*

- *$m_{1}$ and $m_{2}$ fusion provides $m^{\prime }$ = ({0.3000}, {0.1300}, {0.0900}, {0.4800})*
- *$m^{\prime }$ and $m_{3}$ fusion provides m = ({0.4000}, {0.1700}, {0.0900}, {0.3400})*

*Finally, the PPT provides the fused probability distribution.*

$$p\left(A\right)=0.4000+\frac{1}{3}*0.3400=0.5200$$

$$p\left(B\right)=0.1700+\frac{1}{3}*0.3400=0.2800$$

$$p\left(C\right)=0.0900+\frac{1}{3}*0.3400=0.2000$$

*The finally fusion result is p: (0.5200, 0.2800, 0.200).*

**Example** **6.** *Using the data given in Example 1, but the final result is combined by the proposed Beysian update.*

*The corresponding information qualities are:*

$${IQ}_{p_{1}}=0.9800$$

$${IQ}_{P_{2}}=0.9800$$

*The corresponding weightings are:*

$$\omega_{p_{1}}=\omega_{p_{2}}=0.5000$$

*The generated basic probability assignments are:*

$$m_{1}=\left(\left\{0.4950\right\},\left\{0.0050\right\},\left\{0\right\},\left\{0.5000\right\}\right)$$

$$m_{2}=\left(\left\{0\right\},\left\{0.0050\right\},\left\{0.4950\right\},\left\{0.5000\right\}\right)$$

*Next, use of Dempster’s combination rule one time for $m_{1}$ and $m_{2}$ fusion gives m.*

*Fusion of $m_{1}$ and $m_{2}$ obtains m = ({0.3200}, {0.0300}, {0.3200}, {0.3300}).*

*Finally, the PPT provides the fused probability distribution.*

$$p\left(A\right)=0.3200+\frac{1}{3}*0.3300=0.4300$$

$$p\left(B\right)=0.0300+\frac{1}{3}*0.3300=0.1400$$

$$p\left(C\right)=0.3200+\frac{1}{3}*0.3300=0.4300$$

*The final combination result is p: (0.4300, 0.1400, 0.4300).*

Compare the two final combination results (0, 1, 0) and (0.4300, 0.1400, 0.4300); the later result is more reasonable. The high degree conflict example illustrates in this situation that the classical Bayesian method cannot update sensor report but the presented Bayesian method can using evidence theory to provide intuitive updates.

### 4.2. Target Recognition

In this section, an application in target recognition illustrates the efficiency of the proposed method.

Assume three bombs were planted in an area in a military exercise. Three sensors are used to detect the bombs. The data collected from sensors are as follows, $s_{1}$: (0.7000, 0.2000, 0.1000), $s_{2}$: (0.8000, 0.1000, 0.1000), $s_{3}$: (0.6000, 0.2000, 0.2000).

The corresponding information qualities are:

$${IQ}_{s_{1}}=0.5400$$

$${IQ}_{s_{2}}=0.6600$$

$${IQ}_{s_{3}}=0.4400$$

The corresponding weightings are:

$$\omega_{s_{1}}=\frac{0.5400}{0.5400+0.6600+0.4400}=0.3300$$

$$\omega_{s_{2}}=\frac{0.6600}{0.5400+0.6600+0.4400}=0.4000$$

$$\omega_{s_{3}}=\frac{0.4400}{0.5400+0.6600+0.4400}=0.2700$$

The generated basic probability assignments are:

$$m_{1}=\left(\left\{0.2300\right\},\left\{0.0700\right\},\left\{0.0300\right\},\left\{0.6700\right\}\right)$$

$$m_{2}=\left(\left\{0.3200\right\},\left\{0.0400\right\},\left\{0.0400\right\},\left\{0.600\right\}\right)$$

$$m_{3}=\left(\left\{0.1600\right\},\left\{0.0500\right\},\left\{0.0500\right\},\left\{0.7300\right\}\right)$$

Next, use of Dempster’s combination rule gives the following.

- $m_{1}$ and $m_{2}$ fusion gives $m^{\prime }$,
  $$m^{\prime }=\left(\left\{0.4700\right\},\left\{0.0600,\right\}\left\{0.0300\right\},\left\{0.4400\right\}\right)$$
- $m^{\prime }$ and $m_{3}$ fusion gives *m*,
  m=({0.5300},{0.0700},{0.0500},{0.3500})

Finally, the final probability distribution is obtained by PPT.

$$p\left(A\right)=0.5300+\frac{1}{3}*0.3500=0.6500$$

$$p\left(B\right)=0.0700+\frac{1}{3}*0.3500=0.1800$$

$$p\left(C\right)=0.0500+\frac{1}{3}*0.3500=0.1700$$

The final fusion result is *s*: (0.6500, 0.1800, 0.1700).

**Figure 2:**
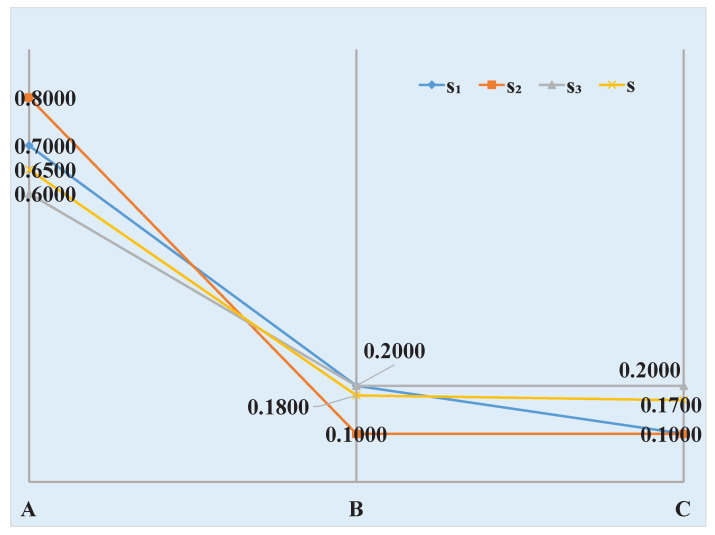
The result of fusion.

From the collected sensor reports, it is easy to know target A is identified, and the fusion result also identifies A, as can be seen in Figure 2.

### 4.3. Multi-Sensor Target Recognition

A real application in multi-sensor target recognition illustrates the virtue of the proposed method compared with the simple average. In a multi-sensor based automatic target recognition system, the detected targets are: A,B,C; suppose the real target is *A*. From five different sensors, the system has collected five bodies of data shown as follows: $s_{1}$ (0.5000, 0.2000, 0.3000), $s_{2}$ (0.7000, 0.1000, 0.2000), $s_{3}$ (0.5500, 0.1000, 0.3500), $s_{4}$ (0.5500, 0.1000, 0.3500), $s_{5}$ (0.6000, 0.1000, 0.3000). The results obtained by the proposed method and simple average are shown in Table 1.

As can be seen from Table 1, when only two collected data simply average, they perform better. However, with collected data increasing the proposed method, better results are achieved compared with simply average. This application shows the proposed method doing better than simply taking the average.

## 5. Conclusions

Bayesian update plays an important role in data fusion. It is reasonable to take information quality into consideration in the Bayesian update process. A new Bayesian update method considering information quality is presented in this paper.

This new way uses discount probability assignment and Dempster’s combination rule. A numerical example and a real application in target recognition illustrate the use of the proposed method. The proposed Bayesian update can deal with conflicting prior probability distributions while the classical Bayesian update cannot.

The main contributions of this paper are mainly in three aspects.

First, it creatively combines information quality with Bayesian update based on the framework of evidence theory.

Second, it proposed a new method to obtain the discount coefficient.

Third, it has the ability to deal with highly conflicting data.

The advantages of the proposed method are as follows: less computation load, strong robustness, fault tolerance. The presented Bayesian update is a generalization of the classical Bayesian update with information quality and conflict taken into account using the framework of evidence theory.

The two open issues and our ongoing works are listed as follows:

One, the input data in this paper is probability distribution. However, in real application of target recognition, the radar report may be modeled by basic probability assignments. As a result, the open issue is to present a new information quality of basic probability assignment.

The other, the proposed method to deal with conflict, depends on the quality of the sensor data report. Determining how to construct the evaluation model, with not only the information in this paper but also the other parameters, is necessary to be considered in future research.

## Figures and Tables

**Table 1:** The results of different combination methods used in multi-sensor target recognition.

	$s_{1}$, $s_{2}$	$s_{1}$, $s_{2}$, $s_{3}$	$s_{1}$, $s_{2}$, $s_{3}$, $s_{4}$	$s_{1}$, $s_{2}$, $s_{3}$, $s_{4}$, $s_{5}$
simple average	p(A)= 0.6000	p(A)= 0.5800	p(A)= 0.5750	p(A)= 0.5800
	p(B)= 0.1500	p(B)= 0.1400	p(B)= 0.1250	p(B)= 0.1200
	p(C)= 0.2500	p(C)= 0.2800	p(C)= 0.3000	p(C)= 0.3000
proposed method	p(A)= 0.5532	p(A)= 0.5924	p(A)= 0.6267	p(A)= 0.6428
	p(B)= 0.1899	p(B)= 0.1490	p(B)= 0.1185	p(B)= 0.1100
	p(C)= 0.2569	p(C)= 0.2586	p(C)= 0.2548	p(C)= 0.2472
